# Supplementary material for: Patterns of Intron Gain and Loss in Fungi
Source: PLoS Biol. 2004 Nov 30;2(12):e422. doi: 10.1371/journal.pbio.0020422 (PMC532390; doi:10.1371/journal.pbio.0020422)
Supplement: Table S1 — Also available at http://genes.mit.edu/NielsenEtAl/. (4.3 MB ZIP). [file pbio.0020422.st001.zip › NielsenEtAl/html/470.html]

AN3655.1.NCU04753.1.MG02490.1.FG09511.1


```
 CLUSTAL W (1.82) Multiple Sequence Alignments - Introns Inserted


Sequence 1: AN3655.1	113 aa
Sequence 2: NCU04753.1	105 aa
Sequence 3: MG02490.1	111 aa
Sequence 4: FG09511.1	116 aa
Alignment Length: 118 aa
Number Identitical Residues: 43 aa
Alignment Score (without introns) 2071


MG02490.1 	MPSIRPSVA-LRALRQTTQT---RTLSTTP--RRLASHGPEYDPPTGWLFGVKPGEKYQK
NCU04753.1	---MPAPTI-LRAGALASR----RAFSTSRAVRSGGAP--HYDPPSGWLFGVRPGEEYKR
FG09511.1 	MALIRAAVAPLRAARCLRQPLSSRGFSVSAR-RQGGGGSHQFDPPTGWLWGVKPGEKAEP
AN3655.1  	MTLRPLSHC-LRARCIRRSAQNIQGFSTRTNLRGGADHGDHYDPPTGYLFGVKPGQKYVK
          	 .    .   ***      . . : :*.    *  .  . .:***:*:*:**:**::   

MG02490.1 	EGWENIMYIGFFGSFAVFTVAMCFKPDTS2IQTWALEEARRRLEVEGILEDPFPEKKP-
NCU04753.1	EGWEIPFFYGFCGSFAVATIAYAFKPDTS2IQTWALEEARRRLEAEGILEDPHPEK---
FG09511.1 	EGWEWPTYI-LLGSLAATAVALAFKPDTT2VSTWALEEARRRLEEEGVLPDPSKQGKKN
AN3655.1  	EGWENIWYYGFIGSLLVAGVAYVFKPDTS2IQTWALEEARRRLEAEGILEDPQRK----
          	****   : .: **: .  :*  *****: :.************ **:* **  :
```
